# Machine learning-based spirometry reference values for the Iranian population: a cross-sectional study from the Shahedieh PERSIAN cohort

**DOI:** 10.3389/fmed.2025.1480931

**Published:** 2025-03-10

**Authors:** Mohammad Sadegh Loeloe, Reyhane Sefidkar, Seyyed Mohammad Tabatabaei, Amir Houshang Mehrparvar, Sara Jambarsang

**Affiliations:** ^1^Center for Healthcare Data Modeling, Department of Biostatistics and Epidemiology, School of Public Health, Shahid Sadoughi University of Medical Sciences, Yazd, Iran; ^2^Department of Medical Informatics, Faculty of Medicine, Mashhad University of Medical Sciences, Mashhad, Iran; ^3^Industrial Diseases Research Center, Department of Occupational Medicine, Shahid Rahnemoon Hospital, Shahid Sadoughi University of Medical Sciences, Yazd, Iran

**Keywords:** spirometry, reference values, respiratory function tests, machine learning, cross-sectional study

## Abstract

**Objective:**

This study aimed to determine spirometric norm values for the healthy Iranian adult population and compare them with established norm equations, specifically the GLI-Caucasian and Iranian equations.

**Methods:**

During the recruitment phase of the Shahedieh Prospective Epidemiological Research Studies in Iran (PERSIAN) in 2016, spirometric parameters of 998 participants were obtained. KNN regression was used to extract reference values for spirometric parameters FEV_1_, FVC, FEV_1_/FVC, and FEF_25–75%_, considering height and age as features. The performance of KNN regression was compared with conventional models used in previous studies, such as the multiple linear regression (MLR) model and the Lambda-Mu-Sigma (LMS) model. The predicted values were compared with those obtained from the GLI-Caucasian and Iranian equations. The validation criterion was the mean squared error (MSE) based on 5-fold cross-validation.

**Results:**

This study included 473 female participants and 525 male participants. KNN regression provided more accurate predictions for four spirometric parameters than MLR and LMS. The MSE for predicting FVC in female participants was 0.159, 0.169, and 0.165 in KNN regression, MLR, and LMS, respectively. The predictions of the present study were closer to the actual values of the reference population for four indicators compared to the prediction values using two sets of reference equations. The MSE of predicted FVC for female participants was 0.159 in the present study, which was less than the Iranian (MSE = 0.344) and GLI-Caucasian (MSE = 0.397) equations.

**Conclusion:**

Using a flexible machine learning approach, this study established spirometry reference values specifically for the Iranian population. Recognizing that spirometry reference values vary among different populations, the Excel calculator developed in this research can be a valuable tool in healthcare centers for assessing lung function in Iranian adults.

## 1 Introduction

The severity and prognosis of respiratory diseases are primarily determined by the results of pulmonary function tests, particularly spirometry (1). Standardized reference values based on population ethnicity, age, and height are necessary for accurate interpretation of spirometry results (2–4). The Global Lung Function Initiative (GLI-2012) in 2012 provided normative reference values for spirometric parameters from over 160,000 samples from 33 countries, which included sex, age, height, and ethnicity-specific equations (4). The suitability of the GLI-2012 equations should be verified before using them for areas not currently covered by the reference equations (5).

The study by Sahebi et al. (6) in Iran showed that the GLI-2012 equations are unsuitable for the Iranian population. The lack of specific predictive values in the Iranian population may lead to disease misclassification, necessitating the standardization of spirometry reference values. Several studies in other populations, for example, Swedish (7), Finnish (8), and Chinese (9), similar to the Iranian study, have recognized that the GLI-2012 equations are not suitable for their populations. However, the appropriateness of the GLI-2012 norms has been confirmed for some populations, e.g., Australian (10), Norwegian (11), German (12), and French (13). As a result, researchers became aware of how crucial it is to identify native reference equations for distinct communities, prompting several studies to identify reference equations for various populations. For instance, in 2017, Jian et al. (1) presented spirometry equations for a Chinese population using the Lambda-Mu-Sigma (LMS) model, Choi et al. (14) developed reference equations for Koreans, Al Qerem et al. (15) presented Jordanian reference values, and Pefura-Yone et al. (16) compared Cameroonian values.

The relationship between spirometry indicators in spirometry tests, such as FEV_1_ and FVC, with age and height variables as independent variables, is non-linear. However, the majority of the previous studies in Iran have used multiple linear regression (MLR) to provide spirometry reference equations. Among these studies, Golshan et al. (17) in Isfahan, Razi et al. (18) in Kashan, Etemadinezhad et al. (19) in Mazandaran, and Aloosh et al. (20) in Hamadan used MLR to provide native reference values for the Iranian population.

The LMS method, which allows simultaneous modeling of the mean (mu), coefficient of variation (sigma), and skewness (lambda) of a distribution family is a special case of the generalized additive model for location, scale, and shape (GAMLSS) and is another widely used method for predicting pulmonary values in spirometry data (21–23). In 2022, Sahebi et al. (6) introduced normal reference equations for Iranians aged 4–82 years, highlighting non-linear predictor–response relationships using LMS and revealing significant differences between Caucasian and Iranian equations.

GAMLSS is a statistical approach used to model data distributions, particularly in fields such as spirometry, where measurements may not conform to traditional assumptions of normality. This method allows for modeling the parameters of a distribution as functions of predictor variables, enabling the modeling of complex relationships in the data. However, implementing and interpreting them can be complicated, and their model selection can be challenging. They can also be computationally intensive, especially with large datasets or when using complex smoothing functions. Overfitting is a risk, and the distribution assumption is crucial in this method.

K-nearest neighbors (KNN) regression is a highly accurate supervised machine learning technique that makes no assumptions about the data distribution. In terms of response prediction, it is more adaptive than linear regression as it derives the model’s structure from the data. This approach overcomes the requirement to verify linearity by supporting non-linear interactions between variables. This non-parametric method has high accuracy in outcome prediction, making it more flexible than linear regression (24, 25). In comparison with GAMLSS, KNN is a method that makes predictions based on the closest training examples in the feature space, while GAMLSS is a parametric model that assumes a specific form for the underlying distribution. Both methods can handle non-linearity but may require larger datasets to estimate parameters and avoid overfitting accurately.

Machine learning (ML) methods, particularly KNN regression, have gained popularity in medical forecasting due to their simplicity and effectiveness. Interpretability and transparency are crucial in healthcare settings. Thus, the fact that machine learning algorithms are incomprehensible makes them a “black box” in many ways. It makes evaluating their efficacy, dependability, and interpretability challenging. To determine generalizability and reliability, clinicians need to know how algorithms produce predictions. For example, SHapley Additive exPlanations (SHAP) values can help healthcare practitioners understand the contributions of individual features to the model’s predictions and provide insight into how different input features influence predicted outcomes, such as lung function based on spirometry data (26). By incorporating population-specific data, models can be tailored to reflect the unique characteristics of the target population, improving the accuracy of predictions (6). By leveraging findings from studies such as Huang et al. (27), practitioners can put relevant features into models such as KNN to accurately forecast health outcomes.

Moreover, this study uses a machine learning method, KNN regression, to predict normal spirometry values for Iranian adults aged 35–70 years. The objective is to provide these values based on age and height features by sex. This study compares the predictions of KNN regression, conventional methods, the MLR model, and the Lambda-Mu-Sigma model with those of Golshan et al.’s equations (17) and the GLI-2012 of the Caucasian reference population (4).

## 2 Methods

### 2.1 Design and participants

This cross-sectional study was nested within the Shahedieh cohort study, in which participants were selected randomly. The details of that study have been published in another study Sabet et al. (28).

### 2.2 Reference population

Of the 2,500 participants in the Shahedieh cohort study for whom a lung test was conducted, 495 (19.8%) people were excluded due to unacceptable maneuvers. Of the remaining 2005 people, after excluding 1,007 people based on other exclusion criteria from the study (Table 1), 998 healthy non-smoker participants (525 male participants and 473 female participants) were included. These exclusion criteria were considered based on several recent studies, which are having sputum cough and rhinorrhea for seven consecutive days, respiratory complaints, history of smoking regularly, history of severe pulmonary disease, physical findings suggestive of cardiopulmonary disease, and evident chest deformity, obesity, and other cases (allergic reactions, occupational conditions, drug use, neurological diseases) (4, 6, 15, 29). In obese people without heart disease, oxygen levels fall as BMI rises. In obesity, correlation with hypoventilation is linked to a decreased residual expiratory volume (30). Therefore, some studies, such as Walid et al. (15), considered a BMI > 30 as an exclusion criterion.

**Table 1:** Reasons for exclusion of participants.

Reasons	*n* = 2,500 (%)
Unacceptable maneuvers	495 (19.8)
Respiratory symptoms	477 (19.1)
Chronic respiratory disease	410 (16.4)
Obesity	428 (17.1)
Tobacco smoking	507 (20.3)
Other	183 (7.3)

### 2.3 Spirometric measurements

Spirometry was performed using the Spirolab III (MIR, Italy), with at least three forced vital capacity (FVC) maneuvers performed for each participant in the morning and in the sitting position, ensuring repeatability and complying with the American Thoracic Society/European Respiratory Society task force (31, 32). Initially, participants unable to perform spirometry maneuvers were identified and excluded from the study. Criteria such as exercising 30 min before the test, eating a large meal within 2 h before the test, and respiratory infections were also considered, and if they were positive, the test was postponed to another time. Then, the maneuver was explained to each participant, and the test was performed under the guidance of the operator. All tests were performed by an operator trained in the spirometry process.

The study measured FVC, forced expiratory volume at 1 s (FEV_1_), FEV_1_/FVC, and forced expiratory flow at 25–75% of FVC (FEF_25–75%_) for each participant. The maneuver with the highest FVC + FEV_1_ was chosen as the best. Demographic and anthropometric variables such as sex, age, height, and weight were recorded. Height was measured without shoes, and age was calculated based on date of birth.

### 2.4 Data analysis

KNN regression is a highly accurate supervised machine learning method that is non-parametric and makes no assumptions about the data distribution. It determines the model’s structure from the data, making it more flexible than linear regression in predicting responses. This method supports non-linear relationships between variables, eliminating the need to check for linearity (24, 25). Average values close together in KNN regression produce estimates that can account for non-linear relationships. By selecting the k-nearest neighborhood, this strategy effectively compensates for the necessity to fit a regression line.

To predict FEV_1_, FVC, FEV_1_/FVC, and FEF_25–75%_ in each sex, KNN regression was used considering age and height as features. Calculations were performed by the Fast Nearest Neighbors (FNN) package (version 1.1.4) in R software (version 4.3.0) (33). To find the optimal K, which is the number of nearest neighbors needed to predict the value of a new data point, we applied 5-fold cross-validation to the training dataset, which provided a good balance between bias and variance (25). There were no significant outliers or missing data in the data preparation stage. To ensure uniform scaling, age and height features were standardized using z-scores. Following data preparation, the available data were initially randomly split into five folds to determine the optimal k (the hyper-parameter) in KNN regression using the five-fold cross-validation (CV) method. The remaining four sections were regarded as the training set, and one of the five was chosen as the test set. The KNN model was trained with the required k-value on the training set by setting the k equal to 1–100. The model was then assessed on the test set, and the mean squared error (MSE) evaluation criterion was computed. To choose each portion as the test set in turn, this procedure was conducted for each of the 5-fold. Finally, the average evaluation results were computed for each k-value. The k-value with the best performance (lowest MSE) in the average assessment results was chosen as the optimal k after the aforementioned procedures were completed for all k-values. Finally, the dataset trained the KNN regression with the optimum k-value. Using this technique, we ensured that the best k was chosen based on how well the model performs on several datasets and not only on a particular data partition. Therefore, the Rfast package (version 2.1.0) was utilized (see Supplementary Figure 1).

This study compared spirometric parameter predictions using KNN regression in the reference population with those from the GLI-2012 study (4), the Caucasian reference population, and the 2003 study by Golshan et al. (17). The final criterion for comparison was the average MSE of 5-fold cross-validation. The agreement between the values predicted by these reference equations was also assessed using the intraclass correlation coefficient (ICC). Finally, the lower limit of the normal (LLN) range was determined for each spirometric parameter, and contour plots were used to display predicted values and LLN simultaneously.

## 3 Results

A total of the 998 participants, the mean (SD) age for male and female participants was 48.4 (7.9) and 45.8 (6.9) years, respectively. The mean (SD) height for male and female participants was 169.7 (7.4) cm and 161.4 (7.7) cm, respectively. Descriptive results for demographic variables and spirometric parameters by sex are depicted in Table 2.

**Table 2:** Demographic and spirometry measurements in the reference population by sex.

Variables	Female participants (*N* = 473)			Male participants (*N* = 525)		
	Mean (SD)	Min.	Max.	Mean (SD)	Min.	Max.
Age (year)	45.81 (6.86)	38	69	48.36 (7.88)	38	69
Height (cm)	161.37 (7.69)	142	185	169.70 (7.40)	138	188
Weight (Kg)	68.28 (9.29)	38	100	74.77 (10.13)	47	106
BMI (Kg/m^2^)	26.19 (2.86)	16	30	25.95 (2.89)	16	30
FEV_1_ (L)	2.56 (0.36)	1.7	3.7	3.47 (0.50)	2.3	4.9
FVC (L)	3.01 (0.45)	2	4.5	4.20 (0.62)	2.8	6.2
FEV_1_/FVC	0.85 (0.04)	0.6	0.9	0.83 (0.05)	0.7	0.9
FEF_25–75%_ (L/s)	3.00 (0.63)	1.5	5.2	3.74 (0.84)	1.9	6.3

Supplementary Table 1 outlines the division of the test data into 5-fold and the presentation of age and height descriptive statistics by sex. Independent *t*-test results show no significant difference between the averages of age and height in each fold and the main data, indicating no significant differences.

### 3.1 Relationship between spirometric parameters and anthropometric features

**Figure 1:**
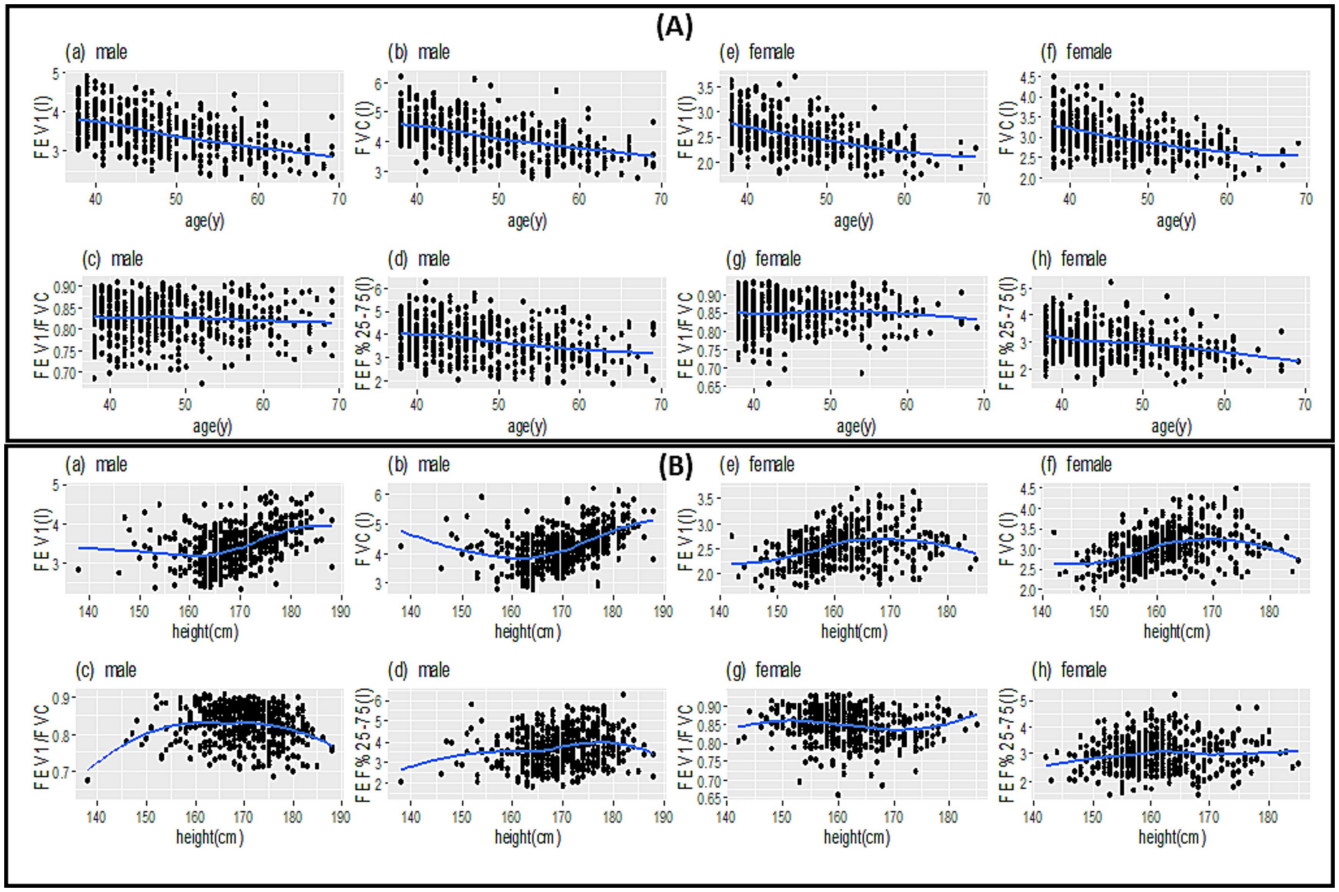
Scatter plot (points in each plot) to examine the relationship between spirometric parameters between age **(A)** and height **(B)** along with the best smooth curve fitted to the data (blue lines).

Figure 1 illustrates the scatter plot of spirometric parameters versus age and height variables by sex and a smoothing curve within each scatter plot. The relationship of spirometric parameters with age is close to a linear relationship (Figure 1A), but the relationship of spirometric parameters with height is close to a non-linear relationship (Figure 1B). Furthermore, based on the smoothed curves, a non-parametric regression can predict the relationship of parameters based on height better than a parametric one (with a specific functional form).

In general, the spirometric parameters of FEV_1_, FVC, and FEF_25–75%_ for male and female participants decrease with an almost constant slope as they age (Figure 1A). The spirometric parameters of FEV_1_ and FVC have a steep increase in male participants with a height above 170 cm. It remains almost constant in female participants with a height above 170 cm. The relationship between FEF_25–75%_ and FEV_1_/FVC with height is more complex for both male and female participants (Figure 1B).

### 3.2 Comparisons

**Figure 2:**
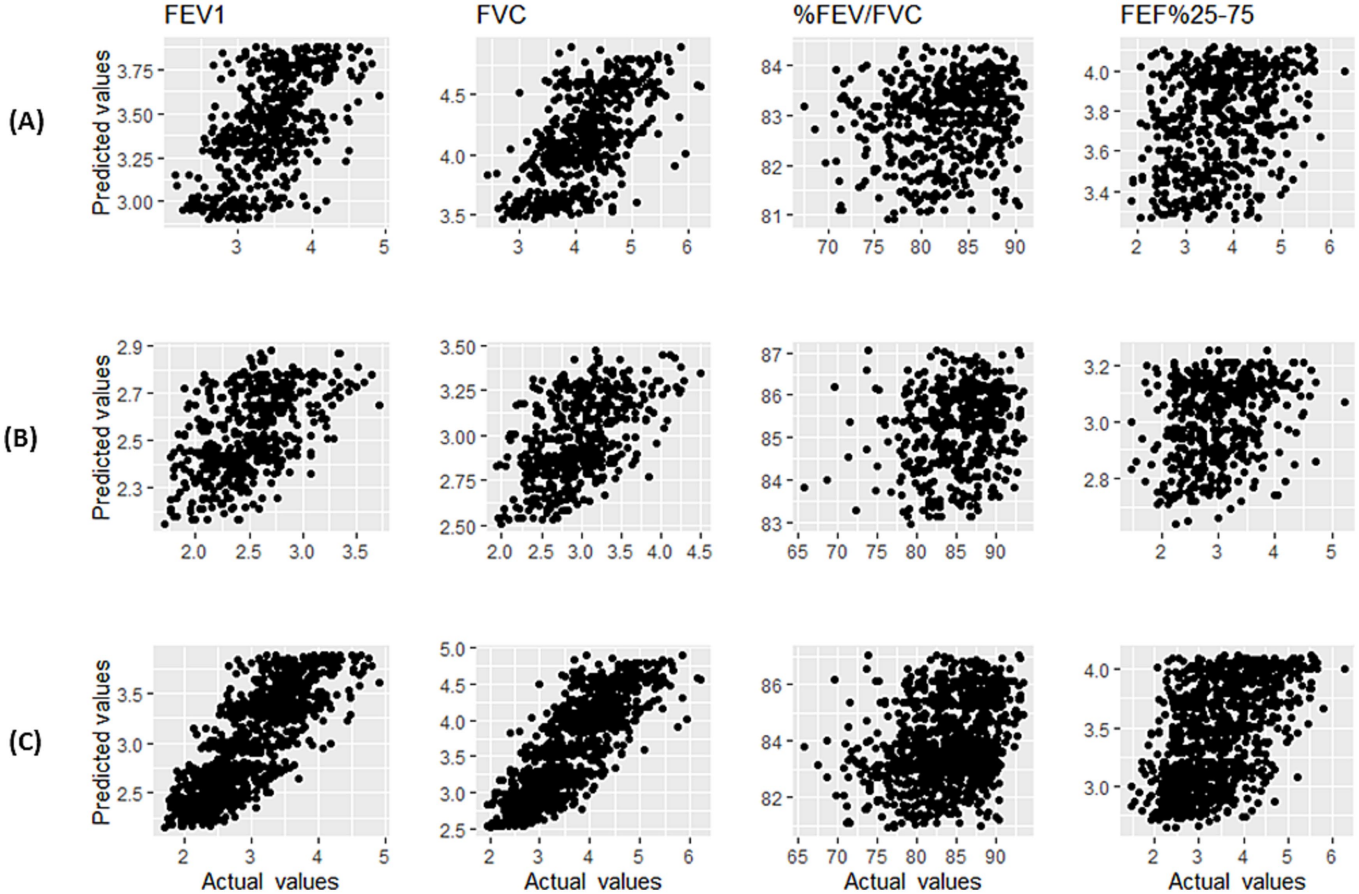
Scatter plot of actual values and predicted values using KNN regression of spirometry indices for male participants **(A)**, female participants **(B)**, and male and female participants **(C)**.

Based on the MSE values presented in Table 3, it can be seen that KNN regression has predicted spirometric parameters based on age and height features closer to the real values than MLR and LMS. The mean (SD) of the predicted spirometric parameters, based on the independent variables of age and height of male and female participants in the reference population of this study, can be observed in Table 4. The comparison of real and predicted values for each spirometric parameter based on paired *t*-tests in all three methods shows that KNN regression has predicted spirometric parameters with less bias. A scatter plot of actual and predicted values with the help of KNN regression based on height and age features and by gender is shown in Figure 2. The points in these graphs are uniformly distributed near the hypothetical 45-degree line and with a balanced dispersion, and no particular pattern can be observed in them.

**Table 3:** Comparison of MSE for KNN, linear regression, and LMS for predicting spirometry parameters of the reference population in this study.

Parameters	Female participants			Male participants		
	KNN	Linear^$^	LMS^#^	KNN	Linear	LMS
FEV_1_	0.1037	0.2817	0.1077	0.1736	0.6550	0.1856
FVC	0.1588	0.1689	0.1649	0.2675	0.2978	0.2994
FEV_1_/FVC	0.1900	0.2000	0.1948	0.2200	0.2300	0.2242
FEF_25–75%_	0.3728	0.3775	0.3766	0.6511	0.6594	0.6586

^#^Fitted model based on the GLI-2012 and other studies: log(Y) = a + b1*log(Height) + b2*log(Age) + M-Spline. ^$^Y = a + b1*Height + b2*Age.

**Table 4:** Mean (SD) for crude and predicted values comparing the MSE of all three methods, our study, Golshan et al. (17), and GLI-2012 (Caucasians) by sex.

Sex	Parameters	Mean (SD)	Predicted						
			Current study			Golshan et al. (17)		Caucasian	
			Mean (SD)	*p*-value	MSE (95% CI)	Mean (SD)	MSE (95% CI)	Mean (SD)	MSE (95% CI)
Female participants	FEV_1_	2.55 (0.39)	2.52 (0.17)	0.452	0.104 (0.10–0.16)	2.82 (0.36)	0.228 (0.17–0.30)	2.82 (0.36)	0.194 (0.17–0.26)
	FVC	3.00 (0.48)	2.96 (0.24)	0.403	0.159 (0.15–0.25)	3.27 (0.43)	0.344 (0.25–0.50)	3.49 (0.44)	0.397 (0.36–0.52)
	%FEV1/FVC	85.42 (4.18)	85.15 (0.96)	0.166	0.190 (0.13–0.24)	86.65 (1.14)	0.224 (0.16–0.33)	81.33 (1.21)	0.296 (0.28–0.36)
	FEF_25–75%_	3.02 (0.66)	2.97 (0.15)	0.113	0.373 (0.33–0.60)	3.64 (0.28)	0.721 (0.57–0.81)	2.86 (0.41)	0.435 (0.33–0.56)
Male participants	FEV_1_	3.43 (0.55)	3.40 (0.28)	0.190	0.174 (0.17–0.28)	3.34 (0.42)	0.330 (0.24–0.40)	3.59 (0.47)	0.300 (0.23–0.39)
	FVC	4.14 (0.68)	4.11 (0.35)	0.261	0.267 (0.24–0.32)	3.89 (0.49)	0.440 (0.34–0.60)	4.50 (0.58)	0.567 (0.40–0.69)
	%FEV1/FVC	82.82 (4.66)	82.94 (0.70)	0.540	0.220 (0.16–0.28)	85.99 (1.00)	0.290 (0.22–0.39)	79.92 (1.25)	0.311 (0.24–0.38)
	FEF_25–75%_	3.74 (0.87)	3.72 (0.23)	0.766	0.651 (0.55–0.87)	4.16 (0.35)	1.26 (0.94–1.49)	3.37 (0.53)	0.887 (0.73–1.19)

In Table 4, the comparison of MSE (and 95% bootstrap confidence interval) based on the predicted values of spirometric parameters for KNN regression and the reference equations of GLI-2012 on the Caucasian population (4) and Golshan et al. (17) is also presented for female and male participants. The MSE values of this study are lower compared to the two other studies, and the confidence intervals were shorter [except for the FEF_25–75%_ of female participants compared to both studies and FEV_1_/FVC of female participants compared to the study of Golshan et al. (17)].

**Figure 3:**
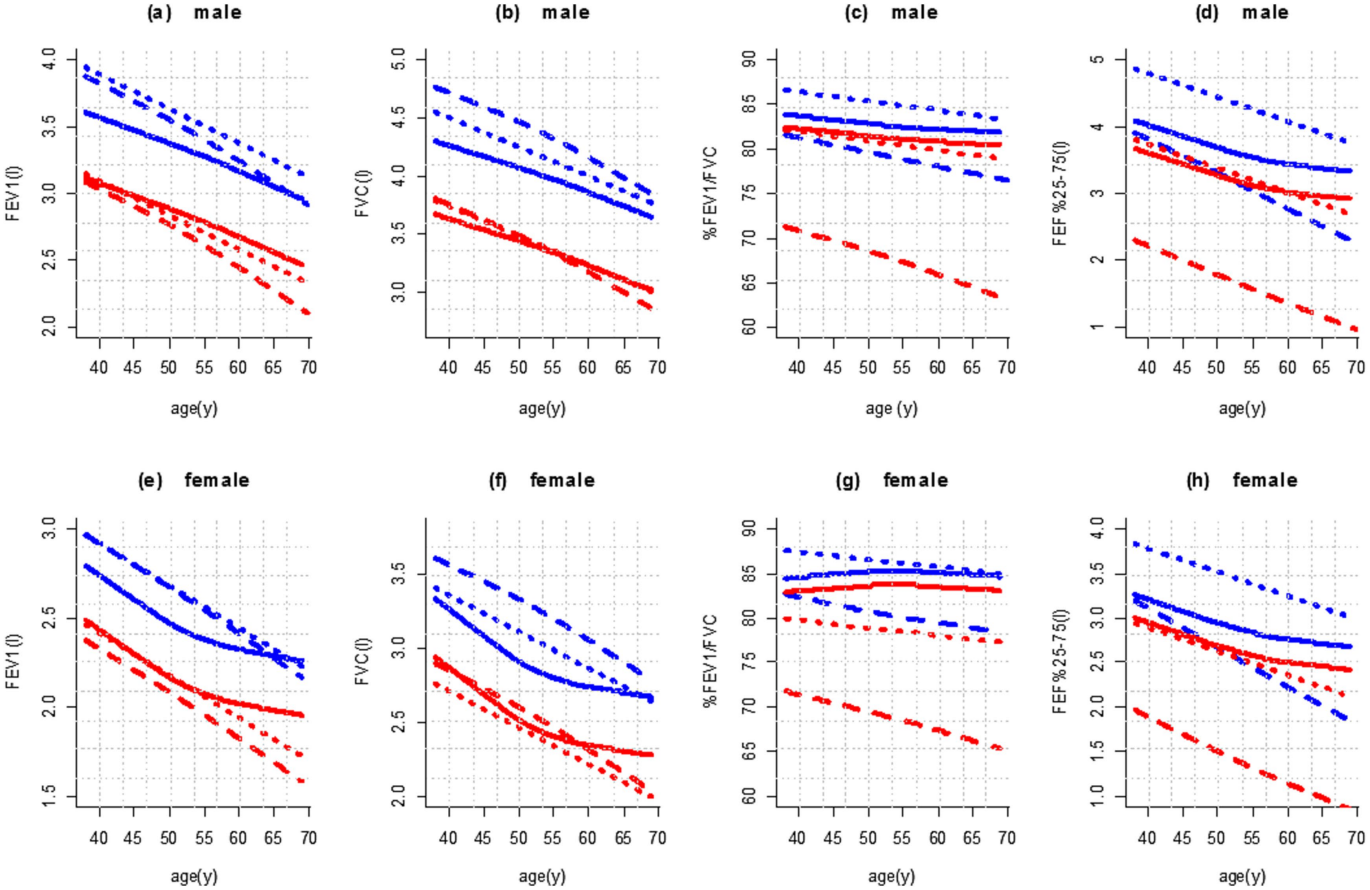
Age dependence of mean values (blue color) and fifth percentile (red color) of spirometry parameters in male (height 170 cm; **a–d**) and female participants (height 160 cm; **e–h**) in comparison with published reference values. Legend: ____: This study; ……: Golshan et al. (17); – – – –: GLI-2012 (Caucasians).

The level of agreement between the three methods was high for predicted values of FEV_1_ (ICC = 0.873, *p* < 0.001) and FVC (ICC = 0.851, *p* < 0.001), but low for FEV_1_/FVC (ICC = 0.154, *p* < 0.001) and moderate for FEF_25–75%_ (ICC = 0.448, *p* < 0.001). This issue can be observed in Figure 3, which shows the prediction of spirometric parameters and LLNs for different ages with height close to the average (160 cm for female participants and 170 cm for male participants) for three methods.

Based on Figures 3a,b, the prediction of FEV_1_ and FVC values with the KNN regression decreased in male participants with almost the same slope with increasing age [and almost parallel to the reference equations of Golshan et al. (17)]. In female participants, from the age of 50 onward, the decline happened with a gentle slope (Figures 3e,f).

### 3.3 Prediction of spirometric parameters based on age and height by sex using KNN regression and the reference population of this study

**Figure 4:**
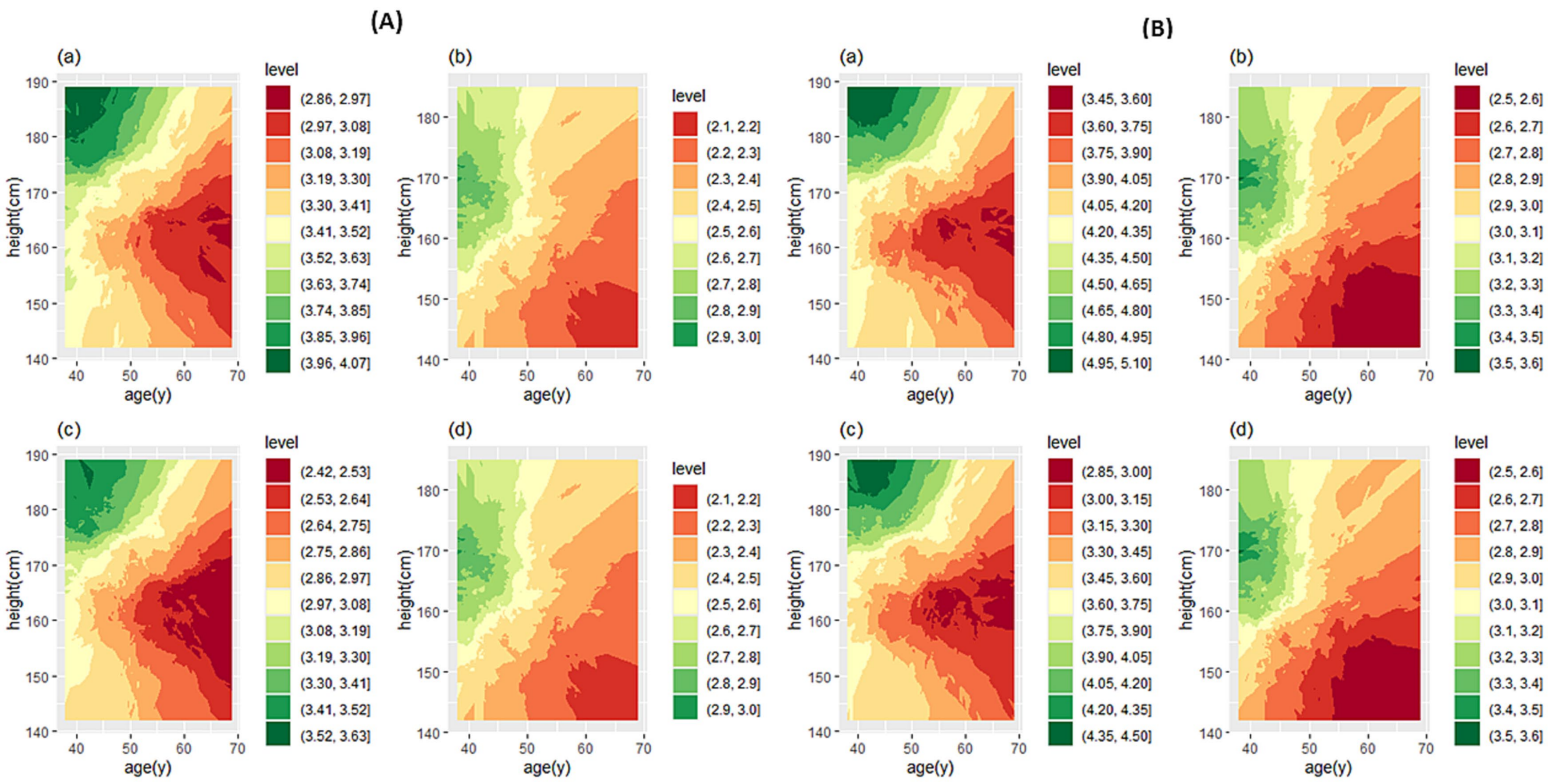
Contour plot for predicted FEV_1_ **(A)** and FVC **(B)**. In each section, the predicted values are for male **(a)** and female participants **(b)**, and the LLN values are for male **(c)** and female participants **(d)**. FEV_1_, forced expiratory volume in 1 s; FVC, forced vital capacity.

**Figure 5:**
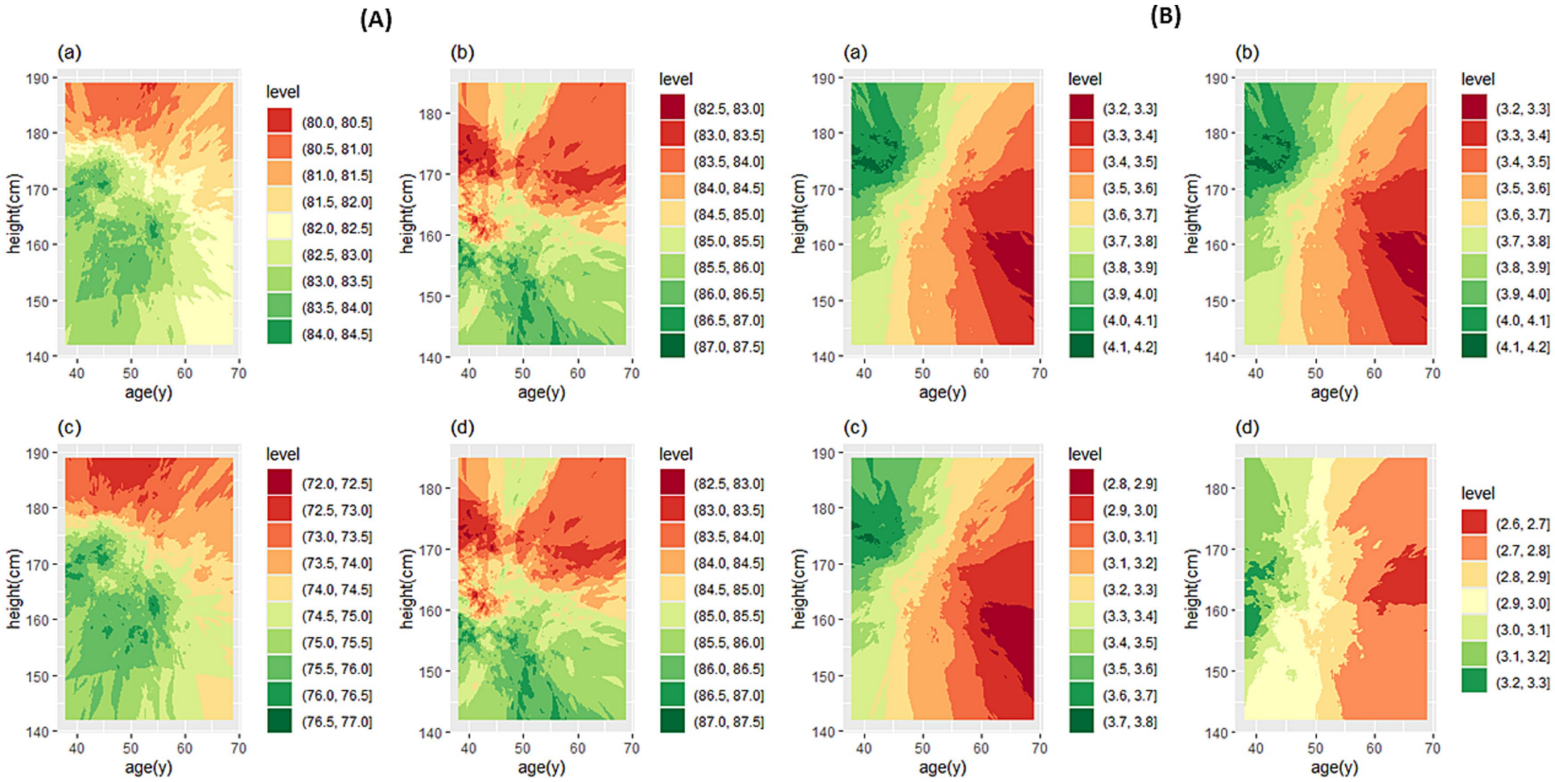
Contour plot for predicted FEV_1_/FVC% **(A)** and FEF_25–75**%**_ **(B)**. In each section, the predicted values are for male **(a)** and female participants **(b)**, and the LLN values are for male **(c)** and female participants **(d)**. FEV_1_ forced expiratory volume in 1 s; FVC forced vital capacity; FEF_25–75%_, forced mid-expiratory flow.

Figure 3 shows the predicted values and the fifth percentile of LLN for spirometric parameters in people aged 35–70 years and height of 160 cm in female participants and 170 cm in male participants. We used the contour plot to access predicted values and LLN more easily, which is shown in Figures 4, 5: FEV_1_ (Figure 4A), FVC (Figure 4B), FEV_1_/FVC% (Figure 5A), and FEF_25–75%_ parameter (Figure 5B).

If the prediction value of a spirometry parameter based on age and height features is needed for a person, contour plots can be used directly. Based on the person’s sex, cross the age (year) from the horizontal axis and the height (cm) from the vertical axis to determine the color range, then based on the specified color with the help of the legend, the numerical value of the parameter can be determined. If spirometry indicators are needed based on a specific range of age or height of people it is suggested to use the website: http://www.graphreader.com/2dreader, which can extract information from colored graphs. If the prediction of spirometry indices based on the features of age and height of a large number of people is required based on the method of this study, the *Excel file* can be used, which is included in Supplementary file 1.

## 4 Discussion

With a machine learning approach, this study was the first to predict normal spirometry values for the Iranian reference population. In this study, we revealed that for the age group of 35–70 years, the relationship of spirometric parameters with age and height is non-linear. As the data showed, the intensity of this non-linear relationship is higher with height. In the study of GLI-2012 (4), the study of Pefura-Yone et al. (16), and a recent study conducted in Iran by Sahebi et al. (6), who used various age groups to provide norm spirometric equations, emphasized the existence of a non-linear relationship between spirometric parameters and age and height. For this reason, the LMS model based on GAMLSS was introduced and used to predict spirometric parameters based on age and height more accurately. Therefore, LMS was proposed as the best model for providing norm spirometry equations in different communities (21, 22). In this study, we used an approach based on machine learning methods to predict the spirometric parameters of the reference population, that is, KNN regression. Compared to MLR and LMS, KNN regression had a lower MSE value for predicting all the spirometric parameters of the reference population, and it was observed that it had more accuracy in predicting the spirometric indices. The prediction of spirometric parameters of the current study was compared to those based on the equations of the common norm in Iran, GLI-2012, and the study of Golshan et al. (17). In the KNN regression method, the average MSE for 5-fold cross-validation showed lower values for all parameters in both male and female participants. It also had shorter bootstrap confidence intervals for most indicators.

Genetic and environmental factors influence the variability of lung function, making it crucial to establish reference values that align with the local population’s ethnic and ecological characteristics (34, 35). Several physiological and environmental elements contribute to the variability between Iranian and Caucasian populations. These variations are influenced by a number of important factors, such as body composition, genetic diversity, and metabolic reactions to nutrition. Iranians may have differing rates of obesity and metabolic syndrome than Caucasian populations because of their historical migrations and connections with numerous ethnic groups. Physiological responses to the climate also influence these variations, which are also influenced by dietary patterns, lifestyle choices, and cultural customs (36, 37).

In contrast to Western diets that are heavy in processed foods and sugars, traditional Iranian diets are rich in grains, legumes, and vegetables and may have different health effects (38). Sedentary lifestyles and urbanization are two examples of lifestyle choices that can affect health consequences. Health behaviors can also be influenced by cultural norms and beliefs on wellbeing, nutrition, and health (39, 40). Finally, health outcomes can also be impacted by the quality and accessibility of healthcare. Addressing public health concerns in these populations requires an understanding of these elements. To this end, we generated prediction values for FEV_1_, FVC, FEV_1_/FVC, and FEF_25–75%_ based on lung function data from 998 participants from healthy Iranian populations, and to present these values and their LLN based on age and height features simultaneously, we used the colored contour plots.

We compared the agreement of lung function predictions between GLI-2012 (Caucasian) (4) and the Iranian population of the current study. The highest agreement was for FEV_1_, and the lowest agreement was for FEV_1_/FVC parameters in male and female participants. Despite the high agreement in the estimation of FEV_1_ and FVC among these three studies, it appears that the Caucasian reference (4) equations and the equations used in the study by Golshan et al. (17) estimated a higher value for the mean and LLN of these two indices than the KNN regression method. However, this difference appears to be more pronounced in individuals under 60 years of age, particularly for FEV_1_. The agreement between the predicted values of FEV_1_/FVC, which were obtained by the KNN regression method, and the study by Golshan et al. (17) was moderate (ICC = 0.481, *p* < 0.001). The agreement was low between the KNN regression method and the Caucasian population (ICC = 0.137, *p* < 0.001) (4). However, based on the three studies, the low agreement between the predicted FEV1/FVC is primarily due to the difference between Caucasian (4) and Iranian equations. The prediction of FEV_1_/FVC for Iranian male and female participants based on the Caucasian reference equations has a lower estimation for the mean and the fifth percentile (LLN) than the method of the present study. The prediction of FEF_25–75%_ for female and male participants based on the Caucasian reference equations (4) has a lower estimation than the KNN regression method. In addition, a high agreement can be seen between the estimation of LLN for FEF_25–75%_ obtained by KNN regression and the reference equations of Golshan et al. (17) (ICC = 0.926, *p* < 0.001), but for female participants over 55 years of age, this difference almost increases. This problem may be caused by menopause as some studies have pointed out a significant decrease in lung function in menopausal women (41, 42). Furthermore, in estimating the average FEF_25–75%_, there is good agreement between the prediction by Caucasian equations (4) and the prediction of values using the KNN regression method for the reference population (ICC = 0.707, *p* < 0.001). However, from the age of 50 years onward for both male and female participants, this difference nearly doubles. It appears that the change in the slope of the relationship between age and these parameters around the age of 50 to 55 is not well explained by a linear model. In general, the KNN regression-based prediction aligned more closely with the Iranian equations of Golshan et al. (17) for all indicators.

K-nearest neighbors (KNN) regression is useful in medical studies but faces several challenges. The choice of K is crucial, as a small K can lead to overfitting, and a large K can smooth out important patterns. In this study, we applied 5-fold cross-validation to the training dataset to find the optimal K that provided a good balance between bias and variance. By using this technique, we can ensure that the best k is chosen based on how well the model performs on several datasets, rather than on a particular data partition (24, 25). Missing data and outliers from several studies can affect KNN regression. Moreover, the features must be on the same scale since KNN regression relies on the distance between points. Consequently, we checked these items during the data preparation step. There were no significant outliers or missing data. To ensure uniform scaling, the features were standardized.

This study has three limitations. Although the sample size of this research is not very large, this sample size of male and female participants is large enough to have sufficient power to validate the spirometric reference values (at least 150 subjects for each sex) (43). The other limitation is that the age range of individuals in this study is 35–70 years, and the predicted reference values can only be used for male and female participants in this age group. Another limitation is that the reference population was selected from only one ethnicity living in Iran (Persian), and other ethnicities were not included in this study.

## 5 Conclusion

It is crucial to determine the norm of pulmonary parameters specific to each population using a suitable model. Therefore, the KNN regression machine learning method was used to predict FEV_1_, FVC, FEV_1_/FVC, and FEF_25–75%_ in a healthy Iranian population of non-smokers aged 35–70 years, based on sex, age, and height. Since the KNN regression method estimates pulmonary parameters with lower MSE, its predicted values could assist physicians in interpreting spirometry results and, when appropriate, in diagnosing diseases and assessing their severity in the Iranian population.

## Data Availability

The original contributions presented in the study are included in the article/Supplementary material, further inquiries can be directed to the corresponding author.
