# Supplementary material for: Machine learning-based spirometry reference values for the Iranian population: a cross-sectional study from the Shahedieh PERSIAN cohort
Source: Front Med (Lausanne). 2025 Mar 10;12:1480931. doi: 10.3389/fmed.2025.1480931 (PMC11938426; doi:10.3389/fmed.2025.1480931)
Supplement: Supplementary file 2 [file Supplementary_file_1.docx]

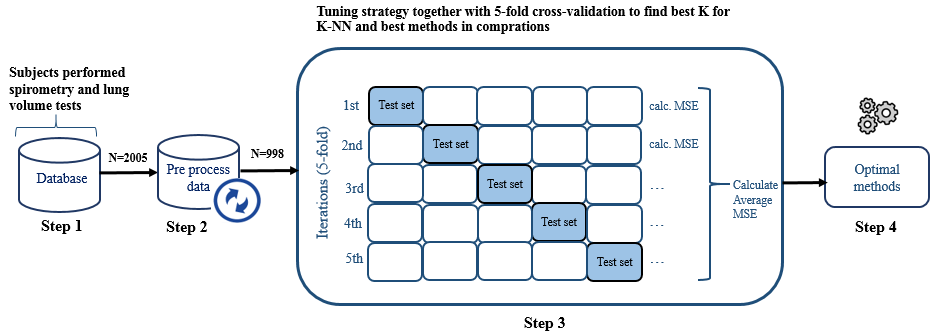


**Figure 1 supplementary;** Illustration of the machine learning-based algorithm for predicting total lung capacity. **MSE**: mean squared error.

| **Table 1 supplementary;** Descriptive statistics of both age (yr.) and height (cm) for 5-folds and comparison by main data. | | | | | | | | |
| --- | --- | --- | --- | --- | --- | --- | --- | --- |
| Gender | Folds | n-Test sets | Covariates | Min | Max | Mean | SD | P-value* |
| Female | Fold 1 | 95 | Age (yr.) | 38 | 69 | 46.4 | 7.8 | 0.33 |
|  |  |  | Height (cm) | 143 | 182 | 162.1 | 7.6 | 0.84 |
|  | Fold 2 | 95 | Age (yr.) | 38 | 67 | 46.5 | 7.4 | 0.58 |
|  |  |  | Height (cm) | 142 | 178 | 161.3 | 7.9 | 0.97 |
|  | Fold 3 | 95 | Age (yr.) | 38 | 69 | 46.0 | 7.3 | 0.24 |
|  |  |  | Height (cm) | 146 | 178 | 161.6 | 6.5 | 0.60 |
|  | Fold 4 | 94 | Age (yr.) | 38 | 69 | 47.6 | 8.2 | 0.13 |
|  |  |  | Height (cm) | 146 | 184 | 162.6 | 8.3 | 0.57 |
|  | Fold 5 | 94 | Age (yr.) | 38 | 67 | 46.2 | 7.0 | 0.44 |
|  |  |  | Height (cm) | 142 | 184 | 160.4 | 8.2 | 0.26 |
|  | Main data | 473 | Age (yr.) | 38 | 69 | 46.6 | 7.5 |  |
|  |  |  | Height (cm) | 142 | 184 | 161.6 | 7.7 |  |
| Male | Fold 1 | 105 | Age (yr.) | 38 | 69 | 48.1 | 8.3 | 0.35 |
|  |  |  | Height (cm) | 157 | 186 | 170.2 | 6.8 | 0.43 |
|  | Fold 2 | 105 | Age (yr.) | 38 | 64 | 47.8 | 7.0 | 0.20 |
|  |  |  | Height (cm) | 147 | 184 | 170.1 | 7.2 | 0.39 |
|  | Fold 3 | 105 | Age (yr.) | 38 | 69 | 48.5 | 7.6 | 0.77 |
|  |  |  | Height (cm) | 148 | 188 | 170.4 | 8.2 | 0.46 |
|  | Fold 4 | 105 | Age (yr.) | 38 | 68 | 49.2 | 8.1 | 0.45 |
|  |  |  | Height (cm) | 138 | 188 | 169.5 | 8.0 | 0.58 |
|  | Fold 5 | 105 | Age (yr.) | 38 | 69 | 50.1 | 7.8 | 0.22 |
|  |  |  | Height (cm) | 138 | 184 | 168.6 | 7.7 | 0.13 |
|  | Main data | 525 | Age (yr.) | 38 | 69 | 48.7 | 7.8 |  |
|  |  |  | Height (cm) | 138 | 188 | 169.8 | 7.6 |  |
| *Mean comparison test (t-test) of each 5-fold with main data | | | | | | | | |
